# Hospital Differences in Cesarean Deliveries in Massachusetts (US) 2004–2006: The Case against Case-Mix Artifact

**DOI:** 10.1371/journal.pone.0057817

**Published:** 2013-03-18

**Authors:** Isabel A. Cáceres, Mariana Arcaya, Eugene Declercq, Candice M. Belanoff, Vanitha Janakiraman, Bruce Cohen, Jeffrey Ecker, Lauren A. Smith, S. V. Subramanian

**Affiliations:** 1 Bureau of Health Information, Statistics, Research, and Evaluation, Massachusetts Department of Public Health, Boston, Massachusetts, United States of America; 2 Department of Social and Behavioral Sciences, Harvard School of Public Health, Boston, Massachusetts, United States of America; 3 Department of Community Health Sciences, Boston University, Boston, Massachusetts, United States of America; 4 Department of Obstetrics and Gynecology, Massachusetts General Hospital, Boston, Massachusetts, United States of America; 5 Massachusetts Department of Health, Boston, Massachusetts, United States of America; University of Vermont, United States of America

## Abstract

**Objective:**

We examined the extent to which differences in hospital-level cesarean delivery rates in Massachusetts were attributable to hospital-level, rather than maternal, characteristics.

**Methods:**

Birth certificate and maternal in-patient hospital discharge records for 2004–06 in Massachusetts were linked. The study population was nulliparous, term, singleton, and vertex births (NTSV) (n = 80,371) in 49 hospitals. Covariates included mother's age, race/ethnicity, education, infant birth weight, gestational age, labor induction (yes/no), hospital shift at time of birth, and preexisting health conditions. We estimated multilevel logistic regression models to assess the likelihood of a cesarean delivery

**Results:**

Overall, among women with NTSV births, 26.5% births were cesarean, with a range of 14% to 38.3% across hospitals. In unadjusted models, the between-hospital variance was 0.103 (SE 0.022); adjusting for demographic, socioeconomic and preexisting medical conditions did not reduce any hospital-level variation 0.108 (SE 0.023).

**Conclusion:**

Even after adjusting for both socio-demographic and clinical factors, the chance of a cesarean delivery for NTSV pregnancies varied according to hospital, suggesting the importance of hospital practices and culture in determining a hospital's cesarean rate.

## Introduction

Cesarean deliveries have been increasing steadily since 1997 in Massachusetts, mirroring the United States (US) trends [1], [2]. In 2009, cesarean births in Massachusetts accounted for 33.6% of all births; a 61% increase from 1998 [3]. Increases in cesarean deliveries have adverse implications for health of the babies, mothers, as well as for health expenditures [4], [5]. Cesarean delivery rates vary widely across states and appear to differ across hospitals. While such differences could reflect differences in the characteristics of hospitals [6], [7], they could also be reflective of differential concentration of maternal characteristics strongly associated with having a cesarean birth across hospitals. For example, efforts to regionalize perinatal care attempt to concentrate potentially high risk births in Level III medical centers [8]. While the question of inter-hospital variation in cesarean rates has received considerable attention outside of the US [9]–[12], few studies have examined this in the US [7], [13] despite findings in other settings that case-mix is unable to completely explain inter-hospital variation in cesarean deliveries [7], [10], [11], [14], [15]. For some fetal conditions, including abnormal fetal heart rate patterns in labor, malpresentation, fetal macrosomia, multiple gestation, and fetal abnormalities, there is some evidence that supports cesarean delivery until better predictive and monitoring tools become available [16]. Ascertaining whether socio-demographic, pregnancy, and clinical characteristics of mothers explain hospital differences in the likelihood of having a cesarean delivery is critical in understanding the specific role of hospitals in contributing to this observed variation. Using a population-based dataset that links birth certificates to hospital discharge data for all births in Massachusetts, we investigate whether hospital variation in cesarean deliveries among a low risk population can be explained by the socio-demographic, pregnancy, and clinical characteristics of mothers.

## Methods

### Data

We used data from the Pregnancy to Early Life Longitudinal (PELL) data system which has linked Massachusetts Birth Certificates (BC) to Hospital Discharge (HD) records since 1998 [17]. We focused on the period of births occurring between January 1, 2004 and December 31, 2006, which includes 228,864 live births to 223,510 resident women delivered in Massachusetts' 49 hospitals with maternity services over the three year period, accounting for 98.2% of all births to Massachusetts' mothers in a Massachusetts' hospital during these years. This period was the most recent with birth certificates linked to hospital discharge records. Maternal socio-demographics, and time of birth were ascertained from the BC, while method of delivery, infant birth weight, gestational age, induction of labor, parity, plurality, non-vertex/malpresentation, and preexisting medical conditions were ascertained from the linked BC and HD data (**Tables S1, S2**).

### Study population and sample size

Of the linked 228,864 BC records, we excluded 3,702 births linked to Birth Defects Surveillance Program (BDSP) records. Of the 225,162 live births with no birth defects, we selected only those births that were nulliparous (first birth), term (37 or more weeks of gestational age), singleton (one offspring), and vertex (head down position) (NTSV) as these are a more homogeneous group at lower risk for cesarean delivery, relevant for the reduction of primary cesarean rates [18], and a good measure of variation related to non-clinical factors [7]. Records with missing maternal education (n = 59) and race (n = 17) and infant birth weight (n = 30) were excluded, with a final analytic sample of 80,265 NTSV births.

### Outcome

Each birth was indicated in the BC records as being delivered through a cesarean procedure or not, which was defined as a binary outcome. This outcome included both planned and emergency cesareans.

### Independent Variables

We considered maternal age in 3 categories: under 30 years, 30 to 34 years, and 35 years and older; educational attainment in 5 categories: less than high school, high school, associated degree, college, and postgraduate; race/ethnicity in 5 categories: White non-Hispanic, Black non-Hispanic, Hispanic, Asian non-Hispanic, and American Indian or other; birth weight of the baby in 3 categories: low (under 2500 g), normal (2500–4000 g), and high (over 4000 g); shift at time of birth in 3 categories: day (8:00AM–7:59PM Monday to Friday, night (8:00PM–7:59AM Monday to Friday), and weekend (Saturday, 8:00AM to Monday, 7:59AM); labor induction (ascertained positive if reported “yes” on BC or on HD with any of the following ICD9 procedure codes: 73.1, 73.01, 73.4), term in 2 categories: early (37–38 weeks of gestational age) and late (39 or more weeks of gestational age); whether the mother was diagnosed (Yes/No) for the following preexisting medical conditions: hypertension, diabetes, eclampsia or pre-eclampsia and placenta previa. The inclusion of these preexisting medical conditions was based on their reporting agreement between birth certificate and hospital discharge records (kappa statistic > = 0.40). Each condition was deemed affirmative if reported on either the birth certificate or hospital discharge (based on ICD9 codes) data sets. All of the above were included as covariates in our analysis (**Table 1**).

**Table 1:** Massachusetts 2004–2006 Nulliparous, Term, Singleton, Vertex (NTSV) Births: Sample Size (n), Percentage Frequency Distribution (%), Percentage of Cesarean Delivery (%CS) and 95% Confidence Interval (CI) by Covariates.

	n	(%)	%CS [95% CI]
Maternal Age (years)			
<30	47,407	(59)	21.7 [21.3,22.1]
30–34	21,948	(27)	29.8 [29.2,30.4]
> = 35	10,910	(14)	41.0 [40.1,41.9]
Maternal Education			
Less than high school	8,721	(11)	18.5 [17.6,19.3]
High school	19,963	(25)	24.5 [23.9,25.1]
Associate degree	16,301	(20)	28.9 [28.2,29.6]
College	21,803	(27)	28.8 [28.1,29.4]
Postgraduate	13,477	(17)	28.3 [27.5,29.1]
Maternal Race and Hispanic Ethnicity			
White non-Hispanic	55,668	(69)	27.5 [27.1,27.8]
Black non-Hispanic	5,988	(7)	29.0 [27.8,30.1]
Hispanic	9,920	(12)	20.8 [20.0,21.6]
Asian	6,662	(8)	25.5 [24.4,26.5]
American Indian, other	2,027	(3)	24.8 [22.9,26.7]
Infant Birth Weight (grams)			
Low (<2500 g)	2,116	(3)	27.3 [25.4,29.2]
Normal (2500–4000 g)	70,736	(88)	24.1 [23.8,24.4]
High (>4000 g)	7,413	(9)	49.3 [48.1,50.4]
Induction of Labor*			
Yes	23,106	(29)	33.2 [32.6,33.8]
No	57,159	(71)	23.8 [23.5,24.2]
Term (weeks of gestational age)			
Early (37–38)	19,378	(24)	24.5 [23.9,25.1]
Late (> = 39)	60,887	(76)	27.2 [26.8,27.5]
Shift at Birth			
Day (M-F, 8:00AM–7:59PM)	29,712	(37)	27.4 [26.9,27.9]
Night (M-F, 8:00PM–7:59AM)	24,587	(31)	25.7 [25.1,26.2]
Weekend (Sat 8:00AM–Mon 7:59AM)	25,966	(32)	26.4 [25.9,26.9]
Pre-existing health risk conditions (No = Reference)			
Hypertension (chronic or gestational)	6,119	(7.6)	37.9 [36.7,39.1]
Diabetes (chronic or gestational)	3,895	(4.9)	41.9 [40.3,43.4]
Eclampsia or pre-eclampsia	3,013	(3.8)	41.3 [39.6,43.1]
Placenta Previa	352	(0.4)	71.9 [67.2,76.6]

* Induction of labor was ascertained positive if reported “yes” on BC or reported on HD with any of the following ICD9 procedure codes: 73.1, 73.01, 73.4.

### Analysis

The data structure for the analysis was hierarchical with births at level-1 (n = 80,265) nested within hospitals at level-2 (n = 49) [19]. The multilevel modeling approach allowed the decomposition of variation in having a cesarean attributable to hospitals, in addition to providing a precision-weighted estimate for hospital-specific predictions [20]. We estimated a multilevel logistic regression to model whether birth 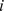 in hospital 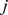 was a cesarean (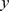) such as 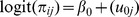 (Model 1) [20], where 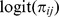 is the log-odds of underlying probability of a cesarean birth 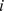 in hospital 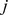. The parameter 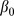 represents the average log-odds of being a cesarean with a random effect (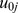) for every hospital. Making identical and independent distribution (IID) assumptions, we estimated a variance at the hospital level-2 (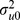) in log-odds of being a cesarean. Results from this model provided a baseline (without adjusting for the characteristics of the mothers) overall variation across hospitals as well as hospital-specific differences (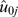). We then extended Model 1 to first include demographic and socioeconomic characteristics of the mothers to the fixed part of the model as 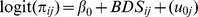 (Model 2), and then the preexisting medical conditions of the mothers as 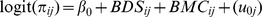 (Model 3). We then assessed the change in the magnitude of the between-hospital variation (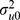) as well as the precision-weighted estimate for hospital-specific differentials (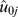) compared to the same estimates from between models 1, 2 and 3. A significant reduction in the hospital variance would suggest that hospital differences largely reflect the clustering of demographic, socioeconomic, and/or preexisting medical conditions of mothers by hospitals. We re-estimated Models 1–3 by considering each of the hospitals as a variable (*i.e.*, a fixed-effect specification) as opposed to being a unit (*i.e.*, a random-effect specification) as a sensitivity test. Models were estimated with the predictive quasi likelihood approximation with second-order Taylor linearization procedure as implemented in MLwiN 2.2 [21].

### Ethical Review

The study was approved by the Massachusetts Department of Public Health Institutional Review Board. The PELL Data System and access to confidential data have been authorized by the Commissioner of Public Health under M.G.L. Chapter 111, Section 24Aand 24B. Each analytic study involving PELL data receives a separate approval from MDPH and from the MA Center for Health Information and Analysis (formerly named MA Division of Health Care Finance and Policy). PELL team members working on specific studies also seek approval from their institutional IRB, including Boston University Medical Center. All data involving confidential identifiers is linked at MDPH on a secure server and de-identified datasets are extracted for analyses. All members of the PELL team sign pledges of confidentiality.

## Results

**Figure 1:**
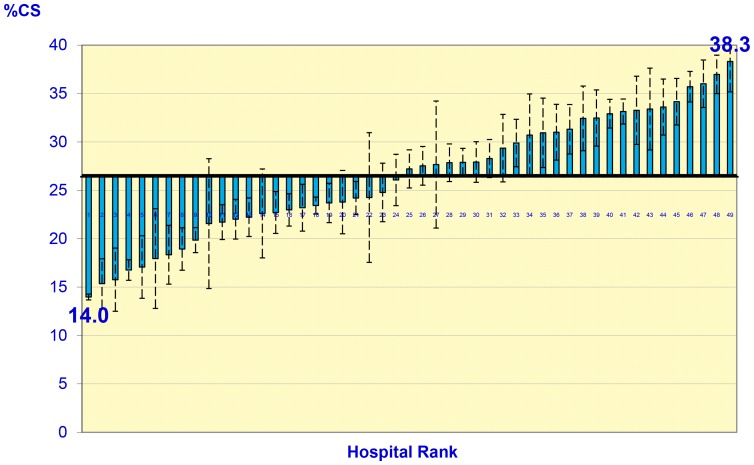
Unadjusted Cesarean Delivery Rates Among Nulliparous, Term, Singleton, Vertex (NTSV) Births in 49 Maternity Hospitals in Massachusetts (US), Massachusetts 2004–2006.

The percentage of cesarean deliveries among Massachusetts nulliparous, term, singleton, and vertex (NTSV) births for 2004–06 was 26.5% (95% CI 26.2, 26.8), and individual risk varied by demographic, socioeconomic, pregnancy, and preexisting medical conditions (**Table 1**). At the hospital level, the percent of cesarean deliveries varied between 14.0% (95% CI 11.4, 16.6) and 38.3% (95% CI 35.4, 41.2), with a mean of 26.4%, which was very similar to the overall cesarean delivery rate among NTSV births (**Figure 1**).

**Figure 2:**
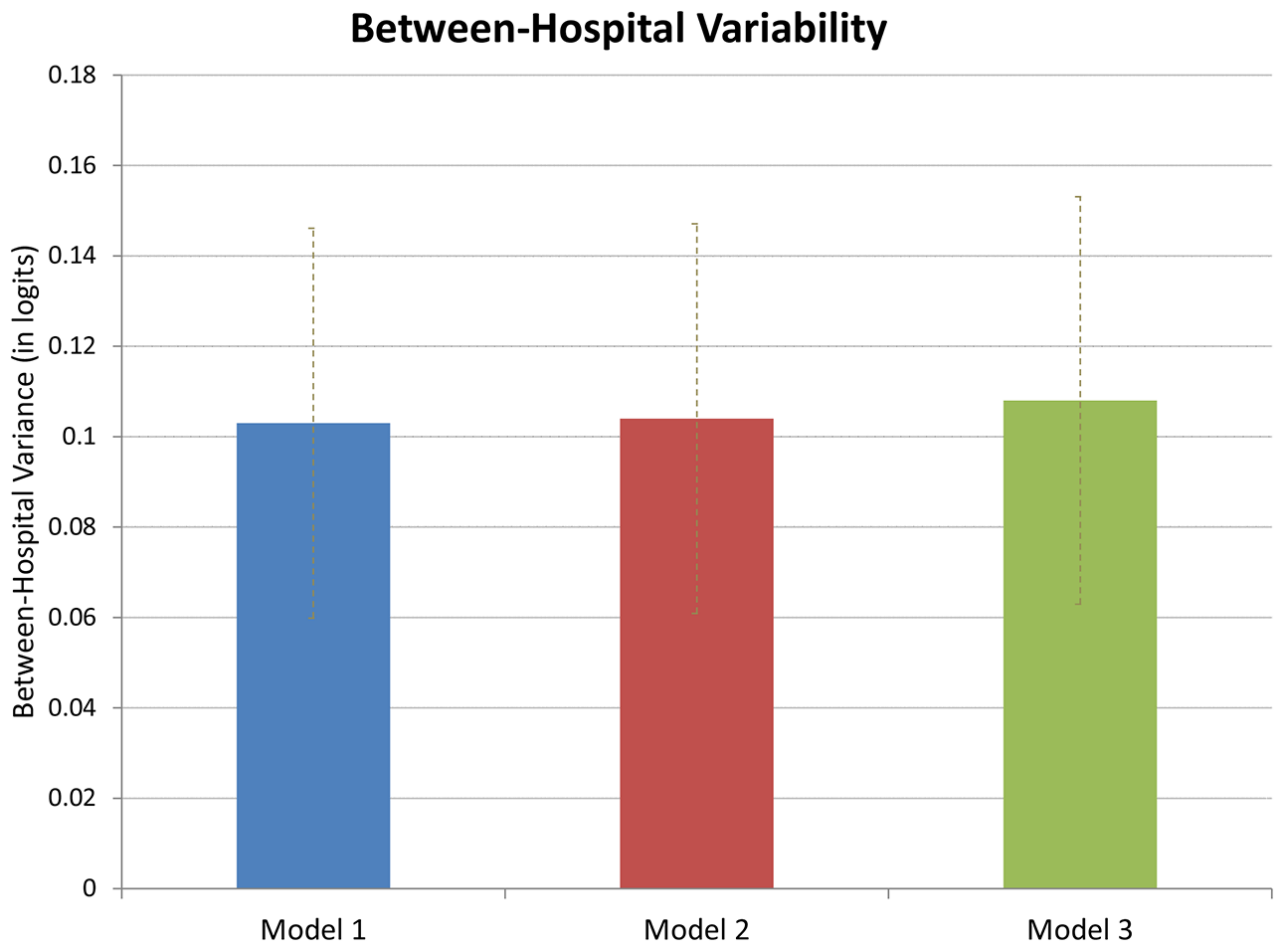
Between-hospital Variation in Cesarean Deliveries with Different Case-mix Adjustment, Massachusetts 2004–2006 NTSV Births.

**Figure 3:**
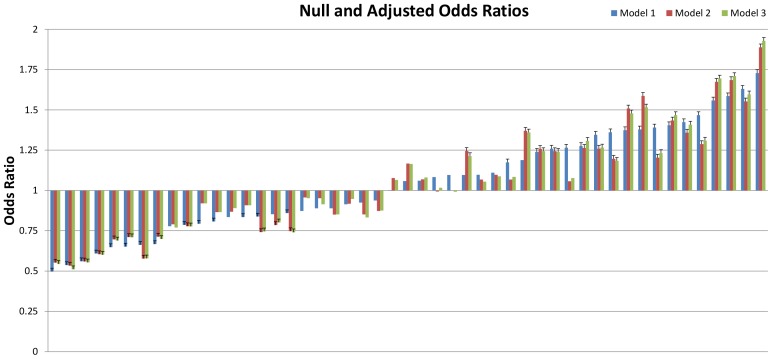
Hospital-specific Odds Ratios of Cesarean Deliveries, Null and Adjusted Models.

In unadjusted models, there was statistically significant variation between hospitals (variance 0.103, SE 0.022) (**Figure 2**). Adjusting for the mother's socio-demographic and pregnancy characteristics did not change this variation (variance 0.103, SE 0.022), and/or additionally adjusting for preexisting medical conditions either one at a time (**Table S3**) or in groups did not alter this variation (variance 0.108, SE 0.023) (**Figure 2**). The hospital-specific differences in cesarean deliveries also remained essentially unchanged between unadjusted and adjusted models (**Figure 3** **, Table S4**). The correlation between hospital-specific ranking across models was very high; 0.95 between Models 1 and 2; 0.956 between Models 1 and 3, and 0.997 between Models 2 and 3.

Reports of labor and delivery complications showed low levels of agreement across birth certificate and hospital discharge records (Kappa <.4), and so were not included in our final models. As a sensitivity analysis, we adjusted Model 3 by adding indicator variables, coded affirmatively if the condition was reported in either the birth certificate or the hospital discharge record, for abruptio placenta, cephalo disproportion, cord prolapse, dysfunctional labor, fever (>100F), fetal distress, rupture of membrane (>24 hr), and meconium. With the exception of fever and ruptured membrane, these predictors were positively associated with cesarean delivery, as expected. Hospital-level variability in cesarean delivery odds actually increased after accounting for these conditions, suggesting that our results were robust and conservative.

We re-estimated Models 1–3 by considering each of the hospitals as a variable (*i.e.*, a fixed-effect specification) and this yielded hospital specific predictions adjusted for case-mix (**Table S5**). The correlation in the predicted probabilities between treating hospitals as a fixed effect (*i.e.*, variables) and as a random effect (*i.e.*, as a unit/level) across the three models was very high (r = 0.99). The associations between a woman's likelihood of cesarean delivery and her socio-demographic characteristics were consistent with those found in previous studies [22]. Placenta previa, eclampsia/preeclampsia, diabetes, hypertension, and high birthweight, were amongst the strongest individual-level clinical predictors of the likelihood of a cesarean delivery (**Table 2**). However, even after adjusting for both socio-demographic and clinical factors, the chance of a cesarean delivery for NTSV pregnancies varied according to hospital.

**Table 2:** Massachusetts 2004–2006 Nulliparous, Term, Singleton, Vertex (NTSV) Births: Adjusted Odds Ratio.

	Adjusted Odds Ratio* [95% CI]
Maternal Age (years)	
<30	Reference
30–34	1.51 [1.45,1.57]
> = 35	2.51 [2.39,2.64]
Maternal Education	
Less than high school	0.89 [0.82,0.96]
High school	1.13 [1.07,1.20]
Associate degree	1.23 [1.16,1.30]
College	1.10 [1.04,1.15]
Postgraduate	Reference
Maternal Race and Hispanic Ethnicity	
White non-Hispanic	Reference
Black non-Hispanic	1.38 [1.29,1.48]
Hispanic	1.00 [0.94,1.06]
Asian	1.02 [0.96,1.09]
American Indian, other	1.12 [1.00,1.24]
Infant Birth Weight (grams)	
Low (<2500 g)	1.13 [1.02,1.25]
Normal (2500–4000 g)	Reference
High (>4000 g)	2.99 [2.84,3.15]
Induction of Labor**	
Yes	1.40 [1.35,1.46]
No	Reference
Term (weeks of gestational age)	
Early (37–38)	Reference
Late (> = 39)	1.21 [1.16,1.26]
Shift at Birth	
Day (M-F, 8:00AM–7:59PM)	1.07 [1.03,1.12]
Night (M-F, 8:00PM–7:59AM)	Reference
Weekend (Sat 8:00AM–Mon 7:59AM)	1.02 [0.98,1.07]
Pre-existing health risk conditions (No = Reference)	
Hypertension (chronic or gestational)	1.39 [1.31,1.47]
Diabetes (chronic or gestational)	1.79 [1.67,1.92]
Eclampsia or pre-eclampsia	1.83 [1.69,1.99]
Placenta Previa	8.22 [6.46,10.47]

* Adjusted for maternal age, education and race, infant birthweight, labor induction, gestational age, shift at birth, and pre-existing medical conditions: diabetes, hypertension, eclampsia, and placenta previa.

** Induction of labor was ascertained positive if reported “yes” on BC or on HD with any of the following ICD9 procedure codes: 73.1, 73.01, 73.4.

## Discussion

Using 2004–06 data from Massachusetts (US), we found that variation across hospitals in the likelihood of a cesarean delivery among a group of lower risk women could not be explained by differences in patient populations with regard to mothers' demographic, socioeconomic, pregnancy-related factors or their preexisting medical conditions.

The association between a women's likelihood of cesarean delivery and her socio-demographic characteristics were consistent with those found in previous studies [23]. Older ages, more educated, Black non-Hispanic women, infants at the extremes of birth weight, induction of labor, over 38 weeks of gestation, births occurring on day shifts, and preexisting medical conditions were associated with an increased risk. Our findings are similar to an Arizona study that found adjusting for maternal risk factors did not explain hospital-level variation in the cesarean delivery rate [7]. Another study of births in military hospitals also found variability in the cesarean delivery rate after adjusting for maternal risk factors [14]. Given the potential risks imposed upon both mother and baby by medically indicated cesarean [17], our findings suggesting that hospital variation persists after case mix-adjustment merits consideration in initiatives focused on lowering the rate of potentially unnecessary cesarean deliveries.

Indeed, some researchers have suggested that the process of decision making in determining the need for cesarean delivery is “almost random” in the US calling for more uniform clinical guidelines on cesarean delivery indications [15]. Hospital level factors noted in previous studies that may influence cesarean delivery rates include: liability- and insurance-related factors [7], [24], [25]; and the presence and type of training program (*e.g.*, whether a woman delivers at a teaching hospital) [7], [14], [26]; Other factors, including the individual physician's approach to delivery [27]–[29], practices related to early hospital admission and pitocin use for labor induction or augmentation [18], and norms regarding involving resident or private services or midwives during labor and delivery [7], [30], [31], may also contribute to a hospital level effect.

A limitation of our analysis is that we were forced to exclude additional labor/delivery complications, including dysfunctional labor and fetal distress, from the analysis due to low data quality.. As we noted earlier, however, results from a sensitivity analysis that included these variables support our conclusions. The low reporting agreement of these complications between BC and HD records (kappa 0.2 and 0.3 for dysfunctional labor and fetal distress, respectively), also reflects the lack of consistent documentation of these already identified controversial indications for cesareans [32].

We attempted to control for maternal factors *within* a hospital by restricting our analysis to NTSV births, a group considered “low risk” for cesarean delivery [7], [18]. It is, however, likely that our estimates of hospital variation are conservative given our sample restriction to NTSV births. The fact that observed covariates at the mother-level did not attenuate the hospital variation supports our findings. Indeed, our focus on examining hospital variation within one state also overcomes the concern related to regional variation in cesarean rates [33].

In summary, observed socio-demographic and health differences among mothers in hospitals across Massachusetts did not explain the substantial hospital-level variation in the likelihood of a cesarean birth among a group of lower risk women. One implication of this finding is that presenting hospital-specific cesarean rates for NTSV births might be appropriate without further case-mix adjustment. Further research on specific modifiable hospital characteristics is needed to identify major drivers of cesarean delivery variability between institutions with the ultimate goal of reducing the influence of non-clinical factors on cesarean delivery risk.

## Supporting Information

Table S1 Prevalence and Percent of Cesarean Delivery for Specific Maternal Demographic and Pregnancy Characteristics by Hospital ID (sorted by level of services), Massachusetts 2004–2006 NTSV Births.
(DOCX)

Table S2 Prevalence and Cesarean Delivery Rates for Maternal Preexisting Health Risk Conditions by Hospital (sorted by level of services), Massachusetts 2004–2006 NTSV Births.
(DOCX)

Table S3 Hospital Variance under Different Types of Case-mix Adjustment, Massachusetts 2004–2006 NTSV Births.
(DOCX)

Table S4 Hospital-specific Differentials (in logits), 95% Confidence Interval and Rank Across 3 Models, Massachusetts 2004–2006 NTSV Births.
(DOCX)

Table S5 Hospital-specific probabilities of having a cesarean delivery from fixed effect (FE) and random effects (RE) models, Massachusetts 2004–2006 NTSV Births.
(DOCX)
