# Supplementary material for: Hospital Differences in Cesarean Deliveries in Massachusetts (US) 2004–2006: The Case against Case-Mix Artifact
Source: PLoS One. 2013 Mar 18;8(3):e57817. doi: 10.1371/journal.pone.0057817 (PMC3601117; doi:10.1371/journal.pone.0057817)
Supplement: Table S1 — Prevalence and Percent of Cesarean Delivery for Specific Maternal Demographic and Pregnancy Characteristics by Hospital ID (sorted by level of services), Massachusetts 2004–2006 NTSV Births. (DOCX) [file pone.0057817.s001.docx]

**Table S1:** Prevalence and Percent of Cesarean Delivery for Specific Maternal Demographic and Pregnancy Characteristics by Hospital ID (sorted by level of services), Massachusetts 2004-2006 NTSV Births

|  |  |  |  | Age >=35 years | | | Associates Degree | | | non-Hispanic Black | | | BWT>4000g | | | Induced Labor | | | Late Term | | | Day Shift | | |
| --- | --- | --- | --- | --- | --- | --- | --- | --- | --- | --- | --- | --- | --- | --- | --- | --- | --- | --- | --- | --- | --- | --- | --- | --- |
| **Level** | **Hospital** | **N** | **%CS** | **n** | **%** | **%CS** | **n** | **%** | **%CS** | **n** | **%** | **%CS** | **n** | **%** | **%CS** | **n** | **%** | **%CS** | **n** | **%** | **%CS** | **n** | **%** | **%CS** |
|  | **Total** | **80,282** | **26.5** | **10,913** | **13.6** | **41.0** | **16,305** | **20.3** | **28.9** | **5,988** | **7.5** | **29.0** | **7,413** | **9.2** | **49.3** | **23,111** | **28.8** | **33.2** | **60,902** | **75.9** | **27.2** | **29,718** | **37.0** | **27.4** |
| I | 2006 | 650 | 23.7 | 95 | 14.6 | 27.4 | 149 | 22.9 | 24.8 | 10 | 1.5 | * | 77 | 11.8 | 44.2 | 169 | 26.0 | 34.3 | 532 | 81.8 | 25.0 | 209 | 32.2 | 20.6 |
| I | 2022 | 478 | 33.3 | 32 | 6.7 | 50.0 | 117 | 24.5 | 41.0 | 22 | 4.6 | 31.8 | 42 | 8.8 | 57.1 | 147 | 30.8 | 44.2 | 382 | 79.9 | 34.3 | 179 | 37.4 | 35.8 |
| I | 2036 | 613 | 17.9 | 33 | 5.4 | 24.2 | 160 | 26.1 | 15.6 | * | 1.5 | * | 62 | 10.1 | 32.3 | 189 | 30.8 | 27.5 | 514 | 83.8 | 17.1 | 216 | 35.2 | 18.5 |
| I | 2042 | 205 | 17.1 | 36 | 17.6 | 13.9 | 59 | 28.8 | 15.3 | * | 2.9 | * | 26 | 12.7 | 34.6 | 64 | 31.2 | 28.1 | 165 | 80.5 | 17.6 | 63 | 30.7 | 17.5 |
| I | 2044 | 157 | 24.2 | 19 | 12.1 | 47.4 | 42 | 26.8 | 19.0 | 19 | 12.1 | * | 12 | 7.6 | * | 67 | 42.7 | 28.4 | 131 | 83.4 | 26.0 | 60 | 38.2 | 33.3 |
| I | 2052 | 136 | 19.9 | 24 | 17.6 | 20.8 | 34 | 25.0 | 26.5 | * | 0.7 | * | 18 | 13.2 | 61.1 | 13 | 9.6 | * | 109 | 80.1 | 22.0 | 54 | 39.7 | 24.1 |
| I | 2061 | 315 | 22.2 | 22 | 7.0 | 22.7 | 62 | 19.7 | 24.2 | 10 | 3.2 | * | 31 | 9.8 | 38.7 | 127 | 40.3 | 27.6 | 247 | 78.4 | 21.9 | 112 | 35.6 | 21.4 |
| I | 2063 | 685 | 33.1 | 45 | 6.6 | 64.4 | 180 | 26.3 | 33.3 | 33 | 4.8 | 45.5 | 60 | 8.8 | 56.7 | 135 | 19.7 | 52.6 | 522 | 76.2 | 35.2 | 268 | 39.1 | 31.7 |
| I | 2082 | 741 | 31.3 | 76 | 10.3 | 56.6 | 207 | 27.9 | 32.9 | 10 | 1.3 | * | 78 | 10.5 | 64.1 | 241 | 32.5 | 46.1 | 605 | 81.6 | 32.2 | 294 | 39.7 | 32.3 |
| I | 2100 | 1,021 | 33.4 | 114 | 11.2 | 47.4 | 270 | 26.4 | 34.1 | 26 | 2.5 | 38.5 | 92 | 9.0 | 66.3 | 273 | 26.7 | 38.8 | 816 | 79.9 | 34.3 | 410 | 40.2 | 34.4 |
| I | 2105 | 989 | 30.9 | 111 | 11.2 | 40.5 | 233 | 23.6 | 30.0 | * | 0.7 | * | 94 | 9.5 | 57.4 | 350 | 35.4 | 35.4 | 743 | 75.1 | 31.5 | 382 | 38.6 | 29.6 |
| I | 2106 | 513 | 16.8 | 50 | 9.7 | 16.0 | 144 | 28.1 | 17.4 | * | 1.4 | * | 54 | 10.5 | 38.9 | 119 | 23.2 | 23.5 | 440 | 85.8 | 16.6 | 187 | 36.5 | 23.0 |
| I | 2108 | 1,669 | 23.4 | 154 | 9.2 | 41.6 | 276 | 16.5 | 22.5 | 265 | 15.9 | 29.4 | 137 | 8.2 | 42.3 | 271 | 16.2 | 37.3 | 1,309 | 78.4 | 24.4 | 552 | 33.1 | 22.1 |
| I | 2114 | 635 | 30.7 | 56 | 8.8 | 32.1 | 143 | 22.5 | 32.2 | 13 | 2.0 | * | 59 | 9.3 | 45.8 | 191 | 30.1 | 35.6 | 504 | 79.4 | 32.3 | 266 | 41.9 | 30.1 |
| I | 2120 | 469 | 15.4 | 51 | 10.9 | 35.3 | 125 | 26.7 | 16.8 | * | 0.6 | * | 45 | 9.6 | 33.3 | 136 | 29.0 | 16.9 | 362 | 77.2 | 15.7 | 153 | 32.6 | 15.0 |
| I | 2127 | 1,194 | 18.3 | 61 | 5.1 | 42.6 | 305 | 25.5 | 22.6 | 44 | 3.7 | 29.5 | 97 | 8.1 | 46.4 | 354 | 29.6 | 23.7 | 945 | 79.1 | 19.3 | 513 | 43.0 | 17.9 |
| I | 2135 | 1,162 | 23.0 | 152 | 13.1 | 40.1 | 339 | 29.2 | 21.8 | 44 | 3.8 | 34.1 | 127 | 10.9 | 45.7 | 412 | 35.5 | 30.3 | 910 | 78.3 | 22.7 | 464 | 39.9 | 20.0 |
| I | 2143 | 445 | 29.9 | 21 | 4.7 | 52.4 | 102 | 22.9 | 34.3 | * | 0.9 | * | 39 | 8.8 | 43.6 | 116 | 26.1 | 39.7 | 375 | 84.3 | 28.5 | 196 | 44.0 | 32.1 |
| I | 2145 | 694 | 14.0 | 56 | 8.1 | 28.6 | 106 | 15.3 | 20.8 | 17 | 2.4 | * | 66 | 9.5 | 22.7 | 181 | 26.1 | 18.8 | 549 | 79.1 | 14.8 | 234 | 33.7 | 15.8 |
| I | 2148 | 178 | 27.5 | 10 | 5.6 | 80.0 | 42 | 23.6 | 38.1 | * | 0.6 | * | 21 | 11.8 | 52.4 | 41 | 23.0 | 41.5 | 151 | 84.8 | 27.2 | 67 | 37.6 | 26.9 |
| I | 2149 | 1,433 | 22.6 | 92 | 6.4 | 50.0 | 414 | 28.9 | 27.1 | 131 | 9.1 | 26.0 | 131 | 9.1 | 45.0 | 407 | 28.4 | 28.3 | 1,143 | 79.8 | 23.3 | 540 | 37.7 | 24.4 |
| I | 2155 | 1,017 | 24.8 | 172 | 16.9 | 39.5 | 249 | 24.5 | 26.1 | 22 | 2.2 | * | 126 | 12.4 | 42.1 | 246 | 24.2 | 32.5 | 824 | 81.0 | 24.8 | 376 | 37.0 | 22.1 |
| I | 2289 | 640 | 28.3 | 71 | 11.1 | 53.5 | 184 | 28.8 | 25.5 | 16 | 2.5 | 43.8 | 74 | 11.6 | 59.5 | 187 | 29.2 | 37.4 | 521 | 81.4 | 29.4 | 218 | 34.1 | 26.6 |
| I | 2313 | 771 | 24.3 | 62 | 8.0 | 43.5 | 211 | 27.4 | 28.0 | 27 | 3.5 | 22.2 | 59 | 7.7 | 49.2 | 362 | 47.0 | 26.2 | 580 | 75.2 | 25.9 | 356 | 46.2 | 23.0 |
| II | 2007 | 2,039 | 27.7 | 312 | 15.3 | 47.4 | 502 | 24.6 | 28.5 | 42 | 2.1 | 28.6 | 238 | 11.7 | 50.0 | 679 | 33.3 | 41.2 | 1,495 | 73.3 | 28.5 | 746 | 36.6 | 31.5 |
| II | 2010 | 1,552 | 21.7 | 77 | 5.0 | 48.1 | 371 | 23.9 | 23.5 | 70 | 4.5 | 12.9 | 81 | 5.2 | 39.5 | 579 | 37.3 | 26.6 | 1,100 | 70.9 | 21.9 | 533 | 34.3 | 19.5 |
| II | 2014 | 1,911 | 27.2 | 248 | 13.0 | 45.2 | 462 | 24.2 | 31.4 | 116 | 6.1 | 25.9 | 154 | 8.1 | 57.1 | 600 | 31.4 | 31.5 | 1,426 | 74.6 | 28.6 | 736 | 38.5 | 29.1 |
| II | 2018 | 1,258 | 31.0 | 288 | 22.9 | 38.2 | 242 | 19.2 | 32.6 | 18 | 1.4 | 38.9 | 125 | 9.9 | 49.6 | 499 | 39.7 | 32.3 | 932 | 74.1 | 31.5 | 552 | 43.9 | 32.1 |
| II | 2020 | 2,244 | 36.0 | 279 | 12.4 | 48.0 | 345 | 15.4 | 37.1 | 85 | 3.8 | 45.9 | 191 | 8.5 | 60.2 | 529 | 23.6 | 44.6 | 1,751 | 78.0 | 36.7 | 865 | 38.5 | 38.0 |
| II | 2040 | 1,914 | 26.1 | 166 | 8.7 | 40.4 | 464 | 24.2 | 28.0 | 91 | 4.8 | 52.7 | 161 | 8.4 | 50.9 | 536 | 28.0 | 32.1 | 1,443 | 75.4 | 28.2 | 693 | 36.2 | 27.4 |
| II | 2058 | 1,482 | 33.6 | 227 | 15.3 | 50.2 | 422 | 28.5 | 36.7 | 89 | 6.0 | 38.2 | 131 | 8.8 | 51.9 | 451 | 30.4 | 38.4 | 1,043 | 70.4 | 33.2 | 525 | 35.4 | 34.7 |
| II | 2071 | 2,413 | 22.7 | 564 | 23.4 | 33.3 | 293 | 12.1 | 25.9 | 92 | 3.8 | 29.3 | 271 | 11.2 | 43.2 | 386 | 16.0 | 32.1 | 1,995 | 82.7 | 23.6 | 848 | 35.1 | 23.0 |
| II | 2075 | 3,478 | 34.2 | 789 | 22.7 | 47.3 | 457 | 13.1 | 35.4 | 66 | 1.9 | 40.9 | 392 | 11.3 | 62.0 | 790 | 22.7 | 32.9 | 2,586 | 74.4 | 35.1 | 1,289 | 37.1 | 37.9 |
| II | 2094 | 2,002 | 27.9 | 304 | 15.2 | 44.1 | 482 | 24.1 | 30.7 | 23 | 1.1 | 43.5 | 198 | 9.9 | 49.0 | 305 | 15.2 | 25.9 | 1,588 | 79.3 | 28.4 | 826 | 41.3 | 29.2 |
| II | 2099 | 1,658 | 22.0 | 98 | 5.9 | 40.8 | 310 | 18.7 | 26.1 | 44 | 2.7 | 27.3 | 107 | 6.5 | 48.6 | 825 | 49.8 | 25.6 | 1,270 | 76.6 | 23.1 | 662 | 39.9 | 20.8 |
| II | 2118 | 1,462 | 35.7 | 90 | 6.2 | 54.4 | 396 | 27.1 | 40.9 | 268 | 18.3 | 36.2 | 133 | 9.1 | 63.9 | 426 | 29.1 | 41.5 | 1,062 | 72.6 | 38.0 | 561 | 38.4 | 39.0 |
| II | 2128 | 1,999 | 21.6 | 193 | 9.7 | 34.7 | 516 | 25.8 | 22.7 | 122 | 6.1 | 22.1 | 210 | 10.5 | 41.0 | 572 | 28.6 | 29.2 | 1,557 | 77.9 | 23.2 | 778 | 38.9 | 21.7 |
| II | 2225 | 1,078 | 38.3 | 97 | 9.0 | 51.5 | 283 | 26.3 | 43.5 | 19 | 1.8 | 42.1 | 103 | 9.6 | 57.3 | 283 | 26.3 | 41.7 | 802 | 74.4 | 39.0 | 442 | 41.0 | 38.2 |
| II | 2311 | 906 | 37.0 | 75 | 8.3 | 56.0 | 251 | 27.7 | 44.2 | 224 | 24.7 | 32.1 | 88 | 9.7 | 65.9 | 365 | 40.3 | 42.5 | 689 | 76.0 | 38.0 | 411 | 45.4 | 37.5 |
| II | 2337 | 1,757 | 27.9 | 113 | 6.4 | 47.8 | 477 | 27.1 | 31.4 | 48 | 2.7 | 20.8 | 114 | 6.5 | 48.2 | 567 | 32.3 | 35.4 | 1,300 | 74.0 | 29.2 | 726 | 41.3 | 28.4 |
| III | 2069 | 5,092 | 32.9 | 1,073 | 21.1 | 45.7 | 691 | 13.6 | 35.2 | 487 | 9.6 | 39.4 | 491 | 9.6 | 56.2 | 1,767 | 34.7 | 40.7 | 3,188 | 62.6 | 33.0 | 1,854 | 36.4 | 34.5 |
| III | 2085 | 1,325 | 29.4 | 223 | 16.8 | 48.4 | 185 | 14.0 | 31.4 | 60 | 4.5 | 31.7 | 91 | 6.9 | 50.5 | 573 | 43.2 | 36.8 | 948 | 71.5 | 30.2 | 481 | 36.3 | 29.7 |
| III | 2107 | 3,822 | 32.5 | 566 | 14.8 | 44.5 | 1,026 | 26.8 | 34.8 | 74 | 1.9 | 41.9 | 524 | 13.7 | 52.1 | 1,274 | 33.3 | 38.5 | 2,964 | 77.6 | 33.5 | 1,490 | 39.0 | 32.0 |
| III | 2124 | 4,542 | 15.8 | 509 | 11.2 | 26.7 | 518 | 11.4 | 16.2 | 276 | 6.1 | 18.8 | 373 | 8.2 | 34.3 | 1,071 | 23.6 | 23.5 | 3,502 | 77.1 | 16.4 | 1,627 | 35.8 | 17.4 |
| III | 2168 | 3,691 | 27.9 | 676 | 18.3 | 42.3 | 589 | 16.0 | 29.5 | 224 | 6.1 | 35.3 | 332 | 9.0 | 49.4 | 1,166 | 31.6 | 38.5 | 2,891 | 78.3 | 29.0 | 1,230 | 33.3 | 28.4 |
| III | 2299 | 996 | 32.4 | 162 | 16.3 | 43.8 | 186 | 18.7 | 31.7 | 123 | 12.3 | 40.7 | 79 | 7.9 | 59.5 | 308 | 30.9 | 38.0 | 727 | 73.0 | 31.9 | 338 | 33.9 | 27.2 |
| III | 2307 | 2,359 | 23.8 | 140 | 5.9 | 43.6 | 551 | 23.4 | 26.9 | 1,007 | 42.7 | 25.7 | 153 | 6.5 | 52.3 | 460 | 19.5 | 34.6 | 1,783 | 75.6 | 25.6 | 773 | 32.8 | 25.6 |
| III | 2339 | 3,549 | 18.9 | 296 | 8.3 | 40.9 | 871 | 24.5 | 22.0 | 355 | 10.0 | 21.4 | 253 | 7.1 | 42.7 | 1,033 | 29.1 | 27.3 | 2,691 | 75.8 | 19.6 | 1,283 | 36.2 | 19.3 |
| III | 2341 | 8,943 | 23.2 | 1,733 | 19.4 | 33.2 | 1,262 | 14.1 | 24.4 | 1,218 | 13.6 | 24.2 | 793 | 8.9 | 43.9 | 2,269 | 25.4 | 25.9 | 6,790 | 75.9 | 23.5 | 3,080 | 34.4 | 23.3 |

**Note**: Cells with less than 10 cases and calculations based on less than 10 cases are suppressed (*) unless the numerator is larger than 4 and the denominator is larger than 30 or the numerator is between 5 and 9 and the denominator is larger than 10.
