# Supplementary material for: Hospital Differences in Cesarean Deliveries in Massachusetts (US) 2004–2006: The Case against Case-Mix Artifact
Source: PLoS One. 2013 Mar 18;8(3):e57817. doi: 10.1371/journal.pone.0057817 (PMC3601117; doi:10.1371/journal.pone.0057817)
Supplement: Table S2 — Prevalence and Cesarean Delivery Rates for Maternal Preexisting Health Risk Conditions by Hospital (sorted by level of services), Massachusetts 2004–2006 NTSV Births. (DOCX) [file pone.0057817.s002.docx]

**Table S2:** Prevalence and Cesarean Delivery Rates for Maternal Preexisting Health Risk Conditions by Hospital (sorted by level of services), Massachusetts 2004-2006 NTSV Births

|  |  |  |  | Hypertension | | | Diabetes | | | Eclampsia | | | Placenta Previa | | |
| --- | --- | --- | --- | --- | --- | --- | --- | --- | --- | --- | --- | --- | --- | --- | --- |
| **Level** | **Hospital** | **N** | **%CS** | **n** | **%** | **%CS** | **n** | **%** | **%CS** | **n** | **%** | **%CS** | **n** | **%** | **%CS** |
|  | **Total** | **80,282** | **26.5** | **6,120** | **7.6** | **37.9** | **3,895** | **4.9** | **41.8** | **3,013** | **3.8** | **41.3** | **352** | **0.4** | **71.9** |
| I | 2006 | 650 | 23.7 | 52 | 8.0 | 36.5 | 14 | 2.2 | 50.0 | 21 | 3.2 | 52.4 | * | 0.8 | * |
| I | 2022 | 478 | 33.3 | 52 | 10.9 | 46.2 | 19 | 4.0 | 42.1 | 27 | 5.6 | 51.9 | * | 0.6 | * |
| I | 2036 | 613 | 17.9 | 42 | 6.9 | 23.8 | 22 | 3.6 | 27.3 | 25 | 4.1 | 32.0 | * | 0.5 | * |
| I | 2042 | 205 | 17.1 | 17 | 8.3 | 35.3 | * | 3.4 | * | * | 3.9 | * | * | * | * |
| I | 2044 | 157 | 24.2 | 30 | 19.1 | 23.3 | * | 2.5 | * | * | 3.8 | * | * | 0.6 | * |
| I | 2052 | 136 | 19.9 | 15 | 11.0 | * | * | 2.9 | * | * | 6.6 | * | * | * | * |
| I | 2061 | 315 | 22.2 | 25 | 7.9 | 40.0 | * | 2.2 | * | * | 2.9 | * | * | * | * |
| I | 2063 | 685 | 33.1 | 51 | 7.4 | 37.3 | 95 | 13.9 | 47.4 | 18 | 2.6 | 27.8 | * | 0.1 | * |
| I | 2082 | 741 | 31.3 | 40 | 5.4 | 57.5 | 18 | 2.4 | 55.6 | 20 | 2.7 | 55.0 | * | 0.3 | * |
| I | 2100 | 1,021 | 33.4 | 78 | 7.6 | 47.4 | 31 | 3.0 | 54.8 | 24 | 2.4 | 37.5 | * | 0.3 | * |
| I | 2105 | 989 | 30.9 | 91 | 9.2 | 37.4 | 42 | 4.2 | 35.7 | 58 | 5.9 | 41.4 | * | 0.3 | * |
| I | 2106 | 513 | 16.8 | 51 | 9.9 | 29.4 | 13 | 2.5 | * | 14 | 2.7 | 42.9 | * | 0.2 | * |
| I | 2108 | 1,669 | 23.4 | 59 | 3.5 | 27.1 | 78 | 4.7 | 35.9 | 52 | 3.1 | 38.5 | 10 | 0.6 | 90.0 |
| I | 2114 | 635 | 30.7 | 60 | 9.4 | 30.0 | 33 | 5.2 | 39.4 | 31 | 4.9 | 41.9 | * | 0.2 | * |
| I | 2120 | 469 | 15.4 | 40 | 8.5 | 20.0 | 23 | 4.9 | 34.8 | 21 | 4.5 | * | * | * | * |
| I | 2127 | 1,194 | 18.3 | 71 | 5.9 | 32.4 | 57 | 4.8 | 29.8 | 44 | 3.7 | 34.1 | * | 0.3 | * |
| I | 2135 | 1,162 | 23.0 | 79 | 6.8 | 34.2 | 40 | 3.4 | 37.5 | 22 | 1.9 | 36.4 | * | 0.6 | * |
| I | 2143 | 445 | 29.9 | 35 | 7.9 | 45.7 | 19 | 4.3 | 47.4 | 24 | 5.4 | 50.0 | * | * | * |
| I | 2145 | 694 | 14.0 | 78 | 11.2 | 17.9 | 39 | 5.6 | 15.4 | 17 | 2.4 | 29.4 | * | * | * |
| I | 2148 | 178 | 27.5 | 13 | 7.3 | * | * | 4.5 | * | * | 3.4 | * | * | * | * |
| I | 2149 | 1,433 | 22.6 | 95 | 6.6 | 37.9 | 74 | 5.2 | 44.6 | 25 | 1.7 | 52.0 | * | 0.5 | * |
| I | 2155 | 1,017 | 24.8 | 81 | 8.0 | 39.5 | 44 | 4.3 | 38.6 | 14 | 1.4 | 50.0 | * | 0.6 | * |
| I | 2289 | 640 | 28.3 | 64 | 10.0 | 31.3 | 25 | 3.9 | 40.0 | 13 | 2.0 | 53.8 | * | 0.9 | * |
| I | 2313 | 771 | 24.3 | 54 | 7.0 | 38.9 | 29 | 3.8 | 51.7 | 22 | 2.9 | 50.0 | * | 0.3 | * |
| II | 2007 | 2,039 | 27.7 | 148 | 7.3 | 39.2 | 70 | 3.4 | 42.9 | 64 | 3.1 | 42.2 | * | 0.3 | * |
| II | 2010 | 1,552 | 21.7 | 130 | 8.4 | 27.7 | 45 | 2.9 | 24.4 | 88 | 5.7 | 36.4 | * | 0.3 | * |
| II | 2014 | 1,911 | 27.2 | 131 | 6.9 | 40.5 | 65 | 3.4 | 46.2 | 57 | 3.0 | 45.6 | 16 | 0.8 | 68.8 |
| II | 2018 | 1,258 | 31.0 | 80 | 6.4 | 42.5 | 80 | 6.4 | 35.0 | 34 | 2.7 | 52.9 | * | 0.4 | * |
| II | 2020 | 2,244 | 36.0 | 167 | 7.4 | 49.7 | 82 | 3.7 | 52.4 | 62 | 2.8 | 64.5 | * | 0.4 | * |
| II | 2040 | 1,914 | 26.1 | 172 | 9.0 | 42.4 | 107 | 5.6 | 36.4 | 66 | 3.4 | 53.0 | * | 0.4 | * |
| II | 2058 | 1,482 | 33.6 | 71 | 4.8 | 54.9 | 62 | 4.2 | 50.0 | 35 | 2.4 | 51.4 | * | 0.5 | * |
| II | 2071 | 2,413 | 22.7 | 143 | 5.9 | 37.1 | 91 | 3.8 | 42.9 | 42 | 1.7 | 42.9 | 10 | 0.4 | 70.0 |
| II | 2075 | 3,478 | 34.2 | 249 | 7.2 | 48.2 | 156 | 4.5 | 44.2 | 81 | 2.3 | 43.2 | 15 | 0.4 | 93.3 |
| II | 2094 | 2,002 | 27.9 | 126 | 6.3 | 47.6 | 118 | 5.9 | 39.8 | 83 | 4.1 | 42.2 | * | 0.3 | * |
| II | 2099 | 1,658 | 22.0 | 90 | 5.4 | 34.4 | 65 | 3.9 | 50.8 | 126 | 7.6 | 41.3 | * | 0.1 | * |
| II | 2118 | 1,462 | 35.7 | 117 | 8.0 | 40.2 | 56 | 3.8 | 51.8 | 53 | 3.6 | 39.6 | * | 0.5 | * |
| II | 2128 | 1,999 | 21.6 | 133 | 6.7 | 27.8 | 126 | 6.3 | 35.7 | 57 | 2.9 | 29.8 | * | 0.2 | * |
| II | 2225 | 1,078 | 38.3 | 54 | 5.0 | 61.1 | 53 | 4.9 | 54.7 | 22 | 2.0 | 68.2 | * | 0.6 | * |
| II | 2311 | 906 | 37.0 | 81 | 8.9 | 50.6 | 43 | 4.7 | 51.2 | 16 | 1.8 | 56.3 | * | 0.3 | * |
| II | 2337 | 1,757 | 27.9 | 233 | 13.3 | 40.8 | 157 | 8.9 | 45.2 | 40 | 2.3 | 50.0 | * | 0.1 | * |
| III | 2069 | 5,092 | 32.9 | 553 | 10.9 | 44.7 | 230 | 4.5 | 57.0 | 114 | 2.2 | 50.0 | 25 | 0.5 | 80.0 |
| III | 2085 | 1,325 | 29.4 | 135 | 10.2 | 43.0 | 58 | 4.4 | 41.4 | 40 | 3.0 | 52.5 | * | 0.6 | * |
| III | 2107 | 3,822 | 32.5 | 481 | 12.6 | 43.0 | 241 | 6.3 | 44.4 | 119 | 3.1 | 49.6 | 10 | 0.3 | 100.0 |
| III | 2124 | 4,542 | 15.8 | 304 | 6.7 | 18.4 | 287 | 6.3 | 27.5 | 285 | 6.3 | 25.6 | 18 | 0.4 | 83.3 |
| III | 2168 | 3,691 | 27.9 | 205 | 5.6 | 36.6 | 152 | 4.1 | 40.8 | 228 | 6.2 | 39.5 | 24 | 0.7 | 75.0 |
| III | 2299 | 996 | 32.4 | 62 | 6.2 | 43.5 | 48 | 4.8 | 50.0 | 59 | 5.9 | 49.2 | * | 0.4 | * |
| III | 2307 | 2,359 | 23.8 | 115 | 4.9 | 29.6 | 99 | 4.2 | 52.5 | 126 | 5.3 | 35.7 | 37 | 1.6 | 21.6 |
| III | 2339 | 3,549 | 18.9 | 199 | 5.6 | 33.2 | 215 | 6.1 | 43.7 | 193 | 5.4 | 34.2 | * | 0.2 | * |
| III | 2341 | 8,943 | 23.2 | 668 | 7.5 | 32.5 | 444 | 5.0 | 37.2 | 393 | 4.4 | 42.2 | 45 | 0.5 | 88.9 |

**Note**: Cells with less than 10 cases and calculations based on less than 10 cases are suppressed (*) unless the numerator is larger than 4 and the denominator is larger than 30 or the numerator is between 5 and 9 and the denominator is larger than 10.
