# Supplementary material for: Hospital Differences in Cesarean Deliveries in Massachusetts (US) 2004–2006: The Case against Case-Mix Artifact
Source: PLoS One. 2013 Mar 18;8(3):e57817. doi: 10.1371/journal.pone.0057817 (PMC3601117; doi:10.1371/journal.pone.0057817)
Supplement: Table S3 — Hospital Variance under Different Types of Case-mix Adjustment, Massachusetts 2004–2006 NTSV Births. (DOCX) [file pone.0057817.s003.docx]

**Table S3:** Hospital Variance under Different Types of Case-mix Adjustment, Massachusetts 2004-2006 NTSV Births

| **Covariate** | **Variance** | **SE** |
| --- | --- | --- |
| Model 1 (no adjustment) | 0.103 | 0.022 |
| Infant Birth Weight | 0.109 | 0.023 |
| Maternal Age | 0.101 | 0.022 |
| Induction | 0.104 | 0.022 |
| Maternal Education | 0.096 | 0.021 |
| Maternal Race | 0.101 | 0.022 |
| Term | 0.105 | 0.023 |
| Delivery Shift | 0.102 | 0.022 |
| Placenta previa | 0.103 | 0.022 |
| Diabetes | 0.104 | 0.022 |
| Hypertension | 0.104 | 0.022 |
| Eclampsia | 0.107 | 0.023 |
| Abruption placenta | 0.103 | 0.022 |
