# Supplementary material for: Hospital Differences in Cesarean Deliveries in Massachusetts (US) 2004–2006: The Case against Case-Mix Artifact
Source: PLoS One. 2013 Mar 18;8(3):e57817. doi: 10.1371/journal.pone.0057817 (PMC3601117; doi:10.1371/journal.pone.0057817)
Supplement: Table S4 — Hospital-specific Differentials (in logits), 95% Confidence Interval and Rank Across 3 Models, Massachusetts 2004–2006 NTSV Births. (DOCX) [file pone.0057817.s004.docx]

**Table S4:** Hospital-specific Differentials (in logits), 95% Confidence Interval and Rank Across 3 Models, Massachusetts 2004-2006 NTSV Births

| **Model 1** | | | | **Model 2** | | | | **Model 3** | | | |
| --- | --- | --- | --- | --- | --- | --- | --- | --- | --- | --- | --- |
| **Hospital** | **Residual** | **95% CI** | **Rank** | **Hospital** | **Residual** | **95% CI** | **Rank** | **Hospital** | **Residual** | **95% CI** | **Rank** |
| 2145 | -0.697 | (-0.912, -0.481) | 1 | 2124 | -0.626 | (-0.749, -0.503) | 1 | 2124 | -0.669 | (-0.794, -0.545) | 1 |
| 2124 | -0.621 | (-0.742, -0.501) | 2 | 2145 | -0.593 | (-0.814, -0.373) | 2 | 2145 | -0.608 | (-0.831, -0.386) | 2 |
| 2120 | -0.577 | (-0.819, -0.336) | 3 | 2120 | -0.580 | (-0.827, -0.332) | 3 | 2120 | -0.590 | (-0.84, -0.341) | 3 |
| 2106 | -0.495 | (-0.724, -0.266) | 4 | 2042 | -0.549 | (-0.869, -0.228) | 4 | 2042 | -0.546 | (-0.87, -0.222) | 4 |
| 2036 | -0.432 | (-0.643, -0.221) | 5 | 2106 | -0.502 | (-0.736, -0.268) | 5 | 2106 | -0.509 | (-0.745, -0.273) | 5 |
| 2127 | -0.427 | (-0.594, -0.26) | 6 | 2036 | -0.361 | (-0.577, -0.144) | 6 | 2036 | -0.371 | (-0.59, -0.153) | 6 |
| 2042 | -0.411 | (-0.725, -0.096) | 7 | 2127 | -0.340 | (-0.511, -0.17) | 7 | 2339 | -0.357 | (-0.484, -0.23) | 7 |
| 2339 | -0.403 | (-0.526, -0.279) | 8 | 2339 | -0.337 | (-0.463, -0.212) | 8 | 2127 | -0.342 | (-0.514, -0.17) | 8 |
| 2052 | -0.250 | (-0.599, 0.1) | 9 | 2071 | -0.298 | (-0.433, -0.164) | 9 | 2341 | -0.299 | (-0.407, -0.191) | 9 |
| 2128 | -0.240 | (-0.379, -0.102) | 10 | 2341 | -0.290 | (-0.397, -0.184) | 10 | 2071 | -0.291 | (-0.427, -0.155) | 10 |
| 2010 | -0.230 | (-0.378, -0.081) | 11 | 2128 | -0.250 | (-0.391, -0.109) | 11 | 2052 | -0.261 | (-0.622, 0.1) | 11 |
| 2099 | -0.213 | (-0.359, -0.068) | 12 | 2135 | -0.241 | (-0.405, -0.077) | 12 | 2128 | -0.251 | (-0.394, -0.108) | 12 |
| 2061 | -0.180 | (-0.436, 0.076) | 13 | 2052 | -0.235 | (-0.593, 0.122) | 13 | 2135 | -0.220 | (-0.385, -0.054) | 13 |
| 2149 | -0.179 | (-0.33, -0.029) | 14 | 2006 | -0.161 | (-0.359, 0.037) | 14 | 2044 | -0.182 | (-0.513, 0.15) | 14 |
| 2071 | -0.176 | (-0.307, -0.045) | 15 | 2044 | -0.160 | (-0.488, 0.168) | 15 | 2006 | -0.160 | (-0.36, 0.041) | 15 |
| 2135 | -0.158 | (-0.318, 0.002) | 16 | 2099 | -0.143 | (-0.293, 0.007) | 16 | 2099 | -0.143 | (-0.295, 0.009) | 16 |
| 2341 | -0.151 | (-0.255, -0.046) | 17 | 2061 | -0.141 | (-0.403, 0.12) | 17 | 2155 | -0.132 | (-0.302, 0.039) | 17 |
| 2108 | -0.136 | (-0.279, 0.008) | 18 | 2155 | -0.136 | (-0.305, 0.032) | 18 | 2061 | -0.115 | (-0.38, 0.149) | 18 |
| 2307 | -0.117 | (-0.248, 0.013) | 19 | 2149 | -0.096 | (-0.25, 0.058) | 19 | 2149 | -0.095 | (-0.251, 0.061) | 19 |
| 2006 | -0.117 | (-0.31, 0.077) | 20 | 2313 | -0.085 | (-0.27, 0.101) | 20 | 2307 | -0.089 | (-0.227, 0.05) | 20 |
| 2313 | -0.089 | (-0.271, 0.092) | 21 | 2010 | -0.083 | (-0.234, 0.069) | 21 | 2010 | -0.083 | (-0.236, 0.07) | 21 |
| 2044 | -0.077 | (-0.399, 0.246) | 22 | 2307 | -0.049 | (-0.185, 0.087) | 22 | 2313 | -0.054 | (-0.241, 0.134) | 22 |
| 2155 | -0.063 | (-0.228, 0.101) | 23 | 2108 | -0.044 | (-0.19, 0.103) | 23 | 2108 | -0.048 | (-0.196, 0.101) | 23 |
| 2040 | 0.002 | (-0.133, 0.138) | 24 | 2007 | -0.006 | (-0.141, 0.129) | 24 | 2168 | -0.007 | (-0.128, 0.113) | 24 |
| 2148 | 0.057 | (-0.245, 0.358) | 25 | 2168 | 0.000 | (-0.118, 0.119) | 25 | 2007 | 0.017 | (-0.119, 0.154) | 25 |
| 2014 | 0.059 | (-0.075, 0.194) | 26 | 2018 | 0.056 | (-0.096, 0.207) | 26 | 2094 | 0.054 | (-0.083, 0.191) | 26 |
| 2007 | 0.081 | (-0.051, 0.214) | 27 | 2085 | 0.066 | (-0.084, 0.216) | 27 | 2040 | 0.064 | (-0.076, 0.204) | 27 |
| 2168 | 0.092 | (-0.024, 0.208) | 28 | 2094 | 0.066 | (-0.069, 0.202) | 28 | 2018 | 0.075 | (-0.078, 0.228) | 28 |
| 2337 | 0.092 | (-0.045, 0.229) | 29 | 2014 | 0.067 | (-0.071, 0.204) | 29 | 2014 | 0.078 | (-0.061, 0.217) | 29 |
| 2094 | 0.094 | (-0.038, 0.227) | 30 | 2040 | 0.075 | (-0.064, 0.213) | 30 | 2085 | 0.082 | (-0.07, 0.234) | 30 |
| 2289 | 0.105 | (-0.082, 0.293) | 31 | 2289 | 0.093 | (-0.099, 0.286) | 31 | 2289 | 0.085 | (-0.109, 0.28) | 31 |
| 2085 | 0.161 | (0.014, 0.308) | 32 | 2148 | 0.155 | (-0.155, 0.466) | 32 | 2148 | 0.153 | (-0.161, 0.467) | 32 |
| 2143 | 0.173 | (-0.039, 0.384) | 33 | 2107 | 0.180 | (0.064, 0.296) | 33 | 2107 | 0.169 | (0.051, 0.287) | 33 |
| 2114 | 0.215 | (0.03, 0.4) | 34 | 2069 | 0.186 | (0.075, 0.298) | 34 | 2337 | 0.194 | (0.052, 0.336) | 34 |
| 2105 | 0.231 | (0.072, 0.39) | 35 | 2105 | 0.219 | (0.056, 0.382) | 35 | 2069 | 0.209 | (0.095, 0.322) | 35 |
| 2018 | 0.236 | (0.088, 0.384) | 36 | 2337 | 0.220 | (0.08, 0.36) | 36 | 2105 | 0.215 | (0.05, 0.379) | 36 |
| 2082 | 0.244 | (0.069, 0.418) | 37 | 2114 | 0.230 | (0.04, 0.419) | 37 | 2114 | 0.219 | (0.028, 0.411) | 37 |
| 2299 | 0.297 | (0.14, 0.455) | 38 | 2299 | 0.231 | (0.07, 0.393) | 38 | 2299 | 0.237 | (0.074, 0.401) | 38 |
| 2107 | 0.309 | (0.195, 0.423) | 39 | 2082 | 0.235 | (0.056, 0.414) | 39 | 2082 | 0.269 | (0.088, 0.45) | 39 |
| 2022 | 0.318 | (0.117, 0.52) | 40 | 2075 | 0.253 | (0.135, 0.371) | 40 | 2075 | 0.270 | (0.151, 0.39) | 40 |
| 2063 | 0.322 | (0.144, 0.499) | 41 | 2058 | 0.307 | (0.165, 0.45) | 41 | 2143 | 0.308 | (0.089, 0.527) | 41 |
| 2069 | 0.330 | (0.221, 0.439) | 42 | 2143 | 0.316 | (0.099, 0.533) | 42 | 2058 | 0.343 | (0.199, 0.487) | 42 |
| 2100 | 0.340 | (0.184, 0.496) | 43 | 2100 | 0.361 | (0.202, 0.52) | 43 | 2100 | 0.384 | (0.223, 0.545) | 43 |
| 2058 | 0.354 | (0.214, 0.493) | 44 | 2022 | 0.412 | (0.205, 0.62) | 44 | 2022 | 0.391 | (0.181, 0.6) | 44 |
| 2075 | 0.384 | (0.269, 0.499) | 45 | 2311 | 0.440 | (0.277, 0.604) | 45 | 2063 | 0.416 | (0.232, 0.601) | 45 |
| 2118 | 0.444 | (0.305, 0.583) | 46 | 2063 | 0.462 | (0.28, 0.644) | 46 | 2311 | 0.468 | (0.303, 0.633) | 46 |
| 2020 | 0.461 | (0.336, 0.586) | 47 | 2118 | 0.516 | (0.373, 0.659) | 47 | 2118 | 0.528 | (0.383, 0.672) | 47 |
| 2311 | 0.489 | (0.33, 0.648) | 48 | 2020 | 0.522 | (0.395, 0.65) | 48 | 2020 | 0.537 | (0.408, 0.666) | 48 |
| 2225 | 0.548 | (0.397, 0.698) | 49 | 2225 | 0.636 | (0.482, 0.79) | 49 | 2225 | 0.657 | (0.501, 0.813) | 49 |

**Note**: Model 1 is unadjusted. Model 2 is adjusted for socioeconomic and demographic factors: maternal age, maternal education, maternal race, infant birth weight, gestational age, labor induction (yes/no), and hospital shift at time of birth. Model 3 is additionally adjusted for the following clinical risk factors: hypertension (chronic or gestational), diabetes (chronic or gestational), eclampsia/pre-eclampsia, and placenta previa.
