# Supplementary material for: Hospital Differences in Cesarean Deliveries in Massachusetts (US) 2004–2006: The Case against Case-Mix Artifact
Source: PLoS One. 2013 Mar 18;8(3):e57817. doi: 10.1371/journal.pone.0057817 (PMC3601117; doi:10.1371/journal.pone.0057817)
Supplement: Table S5 — Hospital-specific probabilities of having a cesarean delivery from fixed effect (FE) and random effects (RE) models, Massachusetts 2004–2006 NTSV Births. (DOCX) [file pone.0057817.s005.docx]

**Table S5:** Hospital-specific probabilities of having a cesarean delivery from fixed effect (FE) and random effects (RE) models, Massachusetts 2004-2006 NTSV Births

| **Hospital** | **Births** | **FE 1** | **RE 1** | **FE 2** | **RE 2** | **FE 3** | **RE 3** |
| --- | --- | --- | --- | --- | --- | --- | --- |
| 2341 | 8943 | 0.232 | 0.232 | 0.100 | 0.100 | 0.090 | 0.090 |
| 2006 | 650 | 0.237 | 0.238 | 0.111 | 0.112 | 0.102 | 0.101 |
| 2007 | 2039 | 0.277 | 0.276 | 0.128 | 0.128 | 0.120 | 0.120 |
| 2010 | 1552 | 0.217 | 0.218 | 0.120 | 0.120 | 0.110 | 0.109 |
| 2014 | 1911 | 0.272 | 0.272 | 0.137 | 0.137 | 0.126 | 0.127 |
| 2018 | 1258 | 0.310 | 0.308 | 0.136 | 0.135 | 0.126 | 0.126 |
| 2020 | 2244 | 0.360 | 0.358 | 0.201 | 0.200 | 0.186 | 0.188 |
| 2022 | 478 | 0.333 | 0.326 | 0.189 | 0.183 | 0.165 | 0.171 |
| 2036 | 613 | 0.179 | 0.186 | 0.091 | 0.094 | 0.084 | 0.082 |
| 2040 | 1914 | 0.261 | 0.260 | 0.138 | 0.138 | 0.125 | 0.125 |
| 2042 | 205 | 0.171 | 0.189 | 0.067 | 0.079 | 0.072 | 0.061 |
| 2044 | 157 | 0.242 | 0.245 | 0.107 | 0.112 | 0.100 | 0.095 |
| 2052 | 136 | 0.198 | 0.215 | 0.096 | 0.105 | 0.093 | 0.085 |
| 2058 | 1482 | 0.336 | 0.333 | 0.169 | 0.168 | 0.159 | 0.160 |
| 2061 | 315 | 0.222 | 0.227 | 0.112 | 0.114 | 0.106 | 0.105 |
| 2063 | 685 | 0.331 | 0.326 | 0.195 | 0.190 | 0.169 | 0.173 |
| 2069 | 5092 | 0.329 | 0.328 | 0.152 | 0.151 | 0.141 | 0.142 |
| 2071 | 2413 | 0.227 | 0.228 | 0.099 | 0.099 | 0.091 | 0.090 |
| 2075 | 3478 | 0.342 | 0.340 | 0.161 | 0.160 | 0.149 | 0.150 |
| 2082 | 741 | 0.313 | 0.310 | 0.160 | 0.158 | 0.149 | 0.151 |
| 2085 | 1325 | 0.294 | 0.292 | 0.137 | 0.137 | 0.127 | 0.127 |
| 2094 | 2002 | 0.279 | 0.279 | 0.137 | 0.137 | 0.124 | 0.124 |
| 2099 | 1658 | 0.220 | 0.221 | 0.113 | 0.114 | 0.104 | 0.103 |
| 2100 | 1021 | 0.334 | 0.330 | 0.178 | 0.175 | 0.164 | 0.167 |
| 2105 | 989 | 0.309 | 0.307 | 0.157 | 0.156 | 0.142 | 0.144 |
| 2106 | 513 | 0.168 | 0.176 | 0.077 | 0.082 | 0.074 | 0.070 |
| 2107 | 3822 | 0.325 | 0.324 | 0.151 | 0.151 | 0.137 | 0.137 |
| 2108 | 1669 | 0.234 | 0.235 | 0.124 | 0.124 | 0.113 | 0.113 |
| 2114 | 635 | 0.307 | 0.303 | 0.160 | 0.157 | 0.143 | 0.145 |
| 2118 | 1462 | 0.357 | 0.354 | 0.201 | 0.199 | 0.185 | 0.187 |
| 2120 | 469 | 0.154 | 0.165 | 0.071 | 0.077 | 0.069 | 0.064 |
| 2124 | 4542 | 0.158 | 0.159 | 0.073 | 0.073 | 0.064 | 0.064 |
| 2127 | 1194 | 0.183 | 0.187 | 0.094 | 0.095 | 0.087 | 0.085 |
| 2128 | 1999 | 0.216 | 0.216 | 0.103 | 0.103 | 0.094 | 0.094 |
| 2135 | 1162 | 0.230 | 0.231 | 0.103 | 0.104 | 0.097 | 0.096 |
| 2143 | 445 | 0.299 | 0.295 | 0.174 | 0.169 | 0.154 | 0.159 |
| 2145 | 694 | 0.140 | 0.149 | 0.071 | 0.076 | 0.068 | 0.064 |
| 2148 | 178 | 0.275 | 0.271 | 0.154 | 0.147 | 0.135 | 0.141 |
| 2149 | 1433 | 0.226 | 0.227 | 0.118 | 0.119 | 0.108 | 0.108 |
| 2155 | 1017 | 0.248 | 0.248 | 0.114 | 0.114 | 0.105 | 0.104 |
| 2168 | 3691 | 0.278 | 0.278 | 0.129 | 0.129 | 0.117 | 0.117 |
| 2225 | 1078 | 0.383 | 0.378 | 0.223 | 0.219 | 0.205 | 0.209 |
| 2289 | 640 | 0.283 | 0.281 | 0.141 | 0.140 | 0.127 | 0.128 |
| 2299 | 996 | 0.324 | 0.321 | 0.159 | 0.157 | 0.145 | 0.147 |
| 2307 | 2359 | 0.238 | 0.238 | 0.124 | 0.124 | 0.109 | 0.109 |
| 2311 | 906 | 0.370 | 0.364 | 0.190 | 0.187 | 0.176 | 0.179 |
| 2313 | 771 | 0.243 | 0.243 | 0.119 | 0.120 | 0.112 | 0.112 |
| 2337 | 1757 | 0.279 | 0.278 | 0.157 | 0.156 | 0.140 | 0.140 |
| 2339 | 3549 | 0.189 | 0.190 | 0.095 | 0.096 | 0.086 | 0.085 |

**Note**: Model 1 is unadjusted. Model 2 is adjusted for sociodemographic and pregnancy factors: maternal age, maternal education, maternal race, infant birth weight, gestational age, labor induction (yes/no), and hospital shift at time of birth. Model 3 is additionally adjusted for the following clinical risk factors: hypertension (chronic or gestational), diabetes (chronic or gestational), eclampsia/pre-eclampsia, and placenta previa.
